# Pituitary Stalk Interruption Syndrome from Infancy to Adulthood: Clinical, Hormonal, and Radiological Assessment According to the Initial Presentation

**DOI:** 10.1371/journal.pone.0142354

**Published:** 2015-11-12

**Authors:** Céline Bar, Charline Zadro, Gwenaelle Diene, Isabelle Oliver, Catherine Pienkowski, Béatrice Jouret, Audrey Cartault, Zeina Ajaltouni, Jean-Pierre Salles, Annick Sevely, Maithé Tauber, Thomas Edouard

**Affiliations:** 1 Endocrine, Bone Diseases, Genetics, Obesity, and Gynecology Unit, Children’s Hospital, University Hospital, Toulouse, France; 2 Neuroradiology Unit, Purpan University Hospital, Toulouse, France; 3 INSERM UMR 1043, Center of Pathophysiology of Toulouse Purpan (CPTP), University of Toulouse Paul Sabatier, Toulouse, France; Erasmus MC, NETHERLANDS

## Abstract

**Background:**

Patients with pituitary stalk interruption syndrome (PSIS) are initially referred for hypoglycemia during the neonatal period or growth retardation during childhood. PSIS is either isolated (nonsyndromic) or associated with extra-pituitary malformations (syndromic).

**Objective:**

To compare baseline characteristics and long-term evolution in patients with PSIS according to the initial presentation.

**Study Design:**

Sixty-seven patients with PSIS were included. Data from subgroups were compared: neonates (n = 10) versus growth retardation patients (n = 47), and syndromic (n = 32) versus nonsyndromic patients (n = 35).

**Results:**

Neonates displayed a more severe hormonal and radiological phenotype than children referred for growth retardation, with a higher incidence of multiple hormonal deficiencies (100% versus 34%; P = 0.0005) and a nonvisible anterior pituitary lobe (33% versus 2%; P = 0.0017). Regular follow-up of growth might have allowed earlier diagnosis in the children with growth retardation, as decreased growth velocity and growth retardation were present respectively 3 and 2 years before referral. We documented a progressive worsening of endocrine impairment throughout childhood in these patients. Presence of extra-pituitary malformations (found in 48%) was not associated with more severe hormonal and radiological characteristics. Growth under GH treatment was similar in the patient groups and did not vary according to the pituitary MRI findings.

**Conclusions:**

PSIS diagnosed in the neonatal period has a particularly severe hormonal and radiological phenotype. The progressive worsening of endocrine impairment throughout childhood justifies periodic follow-up to check for additional hormonal deficiencies.

## Introduction

Pituitary stalk interruption syndrome (PSIS) is characterized by the association of an absent or thin pituitary stalk, an absent or hypoplastic anterior pituitary lobe and/or an ectopic posterior pituitary lobe [1]. This syndrome belongs to the spectrum of midline abnormalities and is often associated with other midline extra-pituitary malformations [2–6]. PSIS is a frequent cause of congenital hypopituitarism and, although the exact prevalence is unknown, abnormal pituitary development has been reported in about half of the patients with congenital hypopituitarism [7]. It is a marker of severe and permanent hormonal impairment [8–11].

To date, the underlying mechanisms involved in PSIS ontogenesis have remained unclear. Perinatal injury was first suspected owing to the high frequency of breech delivery, cesarean section, and neonatal asphyxia in patients with PSIS [12,13]. However, the findings of two thirds of patients without perinatal trauma, familial cases, and the frequent association with extra-pituitary malformations do not fit this hypothesis [5,6,14,15]. The hypothesis of abnormal pituitary development during the embryonic period has more recently been proposed, and mutations of the genes involved in early development (the *HESX1*, *LHX4*, *OTX2*, *SOX3*, and *PROKR2* genes) have indeed been found in patients with congenital hypopituitarism and PSIS. Although this genetic hypothesis is attractive, these gene mutations are nevertheless reported in less than 5% of patients with PSIS, suggesting that most of the genes involved have yet to be discovered [4,14,16].

Patients with PSIS are most frequently referred for the evaluation of growth retardation in childhood. However, the diagnosis can also be made in neonates presenting with hypoglycemia and also in adults [17]. To our knowledge, no study has focused on the neonatal form of PSIS and only a few studies have performed long-term follow-up of the endocrine impairment [18]. In the present study, we report the baseline characteristics, growth hormone response, and long-term evolution of patients with PSIS according to the initial presentation.

## Subjects and Methods

### Patient population

This retrospective longitudinal study comprised all 67 patients (25 females and 42 males) with diagnosed PSIS who were regularly followed in our Endocrinology Unit at the Children’s Hospital in Toulouse, France, between 1984 and 2014.

The diagnosis of PSIS was based on pituitary magnetic resonance imaging (MRI) findings of an absent or thin pituitary stalk associated with at least one of the following radiological features: 1) a nonvisible or hypoplastic anterior pituitary lobe and 2) a nonvisible or ectopic posterior pituitary lobe. The data of these patients were obtained by retrospective chart review.

In line with our study objective, the following patient groups were distinguished. The “neonatal group” consisted of 10 children with PSIS diagnosis stemming from neonatal hypoglycemia, whereas the “growth retardation group” consisted of 47 children with diagnosis stemming from growth retardation. The “syndromic group” consisted of 32 children with PSIS associated with extra-pituitary malformations (in cerebral, ocular, dental, craniofacial, cardiac, or digestive systems), whereas the “nonsyndromic group” consisted of 35 children without an associated malformation.

The study protocol was approved by the Research Ethics Committees of the Purpan University Hospital in Toulouse, France. Written informed consent of the children or their parents was not judged necessary for this kind of retrospective study. Patient information was anonymized and de-identified prior to analysis.

### Personal history and anthropometric measurements

Family history, perinatal characteristics of the patients (including delivery conditions, gestational age, birth measurements, neonatal hypoxemia, hypoglycemia, jaundice, micropenis, and/or cryptorchidism) and associated malformations (including midline abnormalities) were collected.

Birth length, weight, and head circumference were expressed as standard deviation score (SDS) according to Usher and MacLean’s tables [19]. Small for gestational age (SGA) was defined as a birth length and/or weight for gestational age below -2 SDS. An Apgar score below 7 was considered as neonatal distress.

Height, weight, and body mass index (BMI) measurements were converted to age- and sex-specific SDS on the basis of published reference data [20,21].

### Biochemical measurements

Hormones were measured using standard procedures and serum insulin-like growth factor 1 (IGF1) levels were converted to age- and sex-specific SDS, as previously described [22].

Growth hormone deficiency (GHD) was defined as a GH peak of less than 10 μg/L after two stimulation tests. TSH deficiency was defined as a low serum T4 level (< 9 pg/ml) associated with an inappropriate low or normal serum TSH level (< 5 mU/L). ACTH deficiency was defined as a serum cortisol level at 08.00 h less than 70 mg/L associated with an inappropriate low or normal ACTH level (< 60 pg/ml). The gonadotropic axis was tested in patients before 6 months of age (“mini-puberty”) or at postpubertal age (i.e., over 13 years for females and 15 years for males). Gonadotropic deficiency was defined as delayed or absent pubertal development with low serum testosterone or estradiol levels associated with blunted LH/FSH response to a GnRH stimulation test. Prolactin (PRL) deficiency and hyperprolactinemia were defined as basal serum PRL levels less than 5 ng/mL and above 25 ng/mL, respectively. Combined pituitary hormone deficiency (CPHD) was defined as the presence of hormone deficits affecting at least two anterior pituitary hormone lineages, as opposed to isolated GHD (IGHD). When there were clinical findings such as polyuriapolydipsia, a water restriction test was also performed to rule out diabetes insipidus (n = 3).

### Pituitary and brain MRI

MRI was performed with sagittal and coronal T1-weighted sequences and axial T2-weighted sequences. All MRI results were reviewed by the same investigator (CZ) who was not aware of the clinical or endocrine data. Anterior pituitary height was measured and compared with normal values for age [23]. The pituitary stalk was considered to be interrupted when not visible over its entire length, thin when its size was below 1 mm with a very spindly appearance, and absent when not visible at all. Spontaneous hypersignal of the posterior pituitary determined its presence and location. Associated cerebral malformations and midline abnormalities were also sought.

### Follow-up

After starting growth hormone (GH) treatment, children were seen every 6 months for as long as growth continued. Height and weight measurements were obtained at each visit, whereas biochemical measurements were performed every year. Evaluation of the other anterior pituitary functions was performed at diagnosis and repeated during follow-up visits. Twenty-one patients reached their final height and were reevaluated at completion of growth.

### Statistical analyses

Differences between the groups were tested for significance using Mann-Whitney's U-test. Group differences in dichotomous variables were tested for significance using the chi square test. Associations are given as Pearson correlations or Spearman rank correlations, as appropriate.

Multiple regression analysis was used to assess potential predictors of hormonal impairment (coding: IGHD = 1; IGHD and TSH or ACTH deficiency = 2; IGHD and TSH and ACTH deficiency = 3). Gender (coding: male = 1; female = 2), associated extra-pituitary malformations (coding: absence of malformation = 1; presence of malformation = 2), and anterior pituitary abnormality on MRI (coding: anterior pituitary present = 1; anterior pituitary not visible = 2) were introduced as independent variables.

Multiple regression analysis was also used to assess potential predictors of height gain under GH treatment. Age, gender (coding: male = 1; female = 2), height (SDS), hormonal impairment severity (coding: IGHD = 1; IGHD and TSH or ACTH deficiency = 2; IGHD and TSH and ACTH deficiency = 3), and anterior pituitary abnormality on MRI (coding: anterior pituitary present = 1; anterior pituitary not visible = 2) were introduced as independent variables.

The effect of potential predictor variables was assessed in the stepwise mode. All tests were two-tailed and throughout the study P < 0.05 was considered significant. These calculations were performed using SPSS software, version 11.5 for Windows (SPSS Inc., Chicago, IL, USA).

## Results

### General data

Patients were referred for neonatal hypoglycemia (n = 10, 15%), growth retardation (n = 47, 70%), or malformation (n = 10, 15%). All cases were sporadic. There was no documentation of consanguineous parents and no family history of PSIS or CPHD.

As previously described, there was a male predominance with a sex ratio of 1.7 (42 males / 25 females) without difference between groups. Clinical, biochemical, and radiological characteristics were similar in girls and boys and therefore the results of the patients were analyzed as a single group; these features are summarized in Table 1. Median age at diagnosis was 2.5 years (range: from birth to 16.3 years of age) but varied significantly according to the initial presentation, with neonatal and syndromic patients being diagnosed earlier as expected (Table 1). Median height was -2.8 SDS (range: -5.8 to 1.1 SDS) with a bone age retardation of 2.1 years (range: -1.8 to 5.1 years). Evaluation of pretreatment growth curves, available in 33 of 47 children with growth retardation, revealed that growth trajectories started to cross the SDS curves in a downward direction at a median age of 1.0 year (range: 0.3 to 6.0 years), reaching -2 SDS at a median age of 2.0 years (range: 0.5 to 10.2 years), that being two years before the diagnosis was made.

**Table 1:** Clinical and radiological characteristics at baseline of the 67 patients with PSIS and the subgroups.

	PSIS patients	Neonatal	Growth retardation	P	Syndromic	Non-syndromic	P
Number of patients	67	10	47		32	35	
**History**							
Hypoglycemia (%)	25%	100%	14%	< 0.0001	34%	17%	0.1054
Jaundice (%)	22%	30%	16%	0.3005	25%	20%	0.6238
Micropenis (%)	31%	57%	9%	0.0390	42%	22%	0.1553
Cryptorchidism (%)	24%	14%	26%	0.5176	21%	26%	0.7030
**Clinic**							
Age at diagnosis (years)	2.5 (0.0; 16.3)	0 (0; 0.9)	4.1 (0.7; 16.3)	< 0.0001	1.4 (0; 16.3)	4.1 (0; 13.3)	0.0004
Gender (%M/%F)	63% / 37%	70% / 30%	61% / 39%	0.6097	59% / 41%	66% / 34%	0.5920
Height (SDS)	-2.8 (-5.8; 1.1)	-2.2 (-5.2; 1.1)	-2.9 (-5.3; -1.6)	0.1518	-2.9 (-5.8; 0.6)	-2.8 (-5.2; 1.1)	0.7627
Height—TH (SDS)	-2.8 (-6.1; 1.6)	-3 (-6.1; 1.6)	-2.8 (-5.5; -0.9)	0.9057	-2.7 (-5.5; 0.5)	-2.8 (-6.1; 1.6)	0.5082
BMI (SDS)	-0.4 (-3.2; 5.7)	0.2 (-1.2; 2.4)	-0.5 (-3.2; 3.3)	0.0881	-0.3 (-3.2; 5.7)	-0.4 (-3.1; 2.6)	0.5762
**MRI**							
**Pituitary stalk**							
not visible	6%	10%	2%	0.2428	13%	0%	0.0310
interrupted	81%	90%	82%	0.5309	72%	89%	0.0843
thin	13%	0%	16%	0.1764	16%	11%	0.6149
**Anterior pituitary**							
not visible	11%	33%	2%	0.0017	13%	9%	0.5640
hypoplastic	86%	67%	96%	0.0089	83%	88%	0.5735
normal	3%	0%	2%	0.6401	3%	3%	0.9283
**Posterior pituitary**							
not visible	12%	20%	7%	0.1942	19%	6%	0.1002
ectopic	83%	70%	88%	0.1343	81%	86%	0.6222
normal	5%	10%	5%	0.4967	0%	9%	0.0902

Values are medians (range) or percentages as indicated. P values were calculated using the Mann-Whitney test

Concerning neonatal findings, most patients were born full-term (median: 39.7 weeks of gestational age; range: 27 to 42), with only 7 patients born prematurely (< 37 weeks of gestational age). Birth weight was in the normal range (median: -0.2 SDS; range: -2.8 to 2.6 SDS) and birth length was in the lower normal range (median SDS of -0.9; range -3.8 to 2.7) without difference between groups; SGA was found in about 10% of patients. The incidences of breech delivery, cesarean section, and neonatal distress were high (respectively 19, 34, and 26%) without difference between groups.

Features suggestive of neonatal hormonal deficiency (hypoglycemia, jaundice, and/or micropenis/cryptorchidism in males) were found in 28 of 67 patients (42%) among which 14 of the growth retardation group (30%). As expected, male patients with LH/FSH deficiency had a higher incidence of micropenis (60% versus 5%; P = 0.0004) and cryptorchidism (45% versus 5%; P = 0.0067) than patients with a normal gonadotropic axis.

As all patients had GHD, it was not possible to investigate the respective roles of GH and gonadotropic hormones in the origin of genital abnormalities. All patients with hypoglycemia had GHD and ACTH deficiencies.

Although malformation was the presenting symptom in 10 patients, extra-pituitary malformations were found in 32 patients (48%) after initial evaluation, especially in the central nervous system (n = 16, 50%) and the craniofacial structures (n = 11, 34%) (Table 2).

**Table 2:** Characteristics of patients with extra-pituitary malformations (syndromic group).

Gender	Age at diagnosis (years)	Associated malformations	Molecular analysis
M	0.0	Agenesis of corpus callosum	
M	0.0	ASD	OTX2, LHX4 negative
M	0.1	Incisor agenesis, nasal pyriform aperture stenosis	
M	0.1	ASD	
M	0.1	Incisor agenesis, nasal pyriform aperture stenosis, bicuspidia, iris coloboma, VSD	LHX4, HESX1, PROKR2 negative
M	0.2	Agenesis of corpus callosum, frontal encephalomeningocele, iris coloboma	SOX3, HESX1, LHX4 negative
F	0.4	Right frontal cortical dysplasia, VSD	
M	0.5	Mega cisterna magna	
F	0.5	White matter heterotopia, Chiari I malformation	LHX4 negative
M	0.7	Ventricular dilatation, bifid tongue, frontal angioma	SOX3 negative
F	0.7	Incisor agenesis	LHX4 negative
F	0.8	Lobar holoprosencephaly, absent septum pellucidum, hydrocephalus	HESX1 negative
F	0.8	Optic nerve and chiasm hypoplasia, nodular gray matter heterotopia	HESX1, LHX4, OTX2, SOX2 negative
F	1.2	Optic nerve and chiasm hypoplasia, SOD, gray matter heterotopia, VSD	HESX1 negative
M	1.2	Right optic nerve hypoplasia	HESX1, LHX4, OTX2 negative
F	1.3	Left optic nerve hypoplasia	HESX1 negative
M	1.3	Optic nerve and chiasm hypoplasia	HESX1, SOX2 negative
M	1.4	Ogival palate, ocular hypertelorism	RSK2, SOX3, CGH array negative
M	1.6	Chiari I malformation, incisor agenesis, nasal pyriform aperture stenosis	
M	1.9	Optic nerve and chiasm hypoplasia, arachnoid cyst	HESX1, PIT1, OTX2, FMR1 negative
F	1.9	Chiari I malformation	
F	2.3	Fusion of right lateral semi-circular canal with vestibule	15q24 microdeletion
M	2.4	Rectal duplication	TW1, LHX4, SOX3, HESX1 negative
F	2.5	Transsphenoidal encephalocele, cleft palate	HESX1 negative
F	3.5	VSD, anal stenosis	Tetrasomy 22pter-q11.1
M	3.8	Blepharophimosis ptosis epicanthus inversus syndrome	
M	3.8	Retrocerebellar arachnoid cyst, mammillary tubercle hyperplasia	SOX3, HESX1, LHX4 negative
F	7.8	Incisor agenesia	HESX1, LHX4, OTX2, PIT1 negative
F	8.8	Mega cisterna magna, cerebellar vermian atrophy, atrial septal defect	Trisomy 12 mosaicism
M	11.4	Cleft lip palate, nasal hypoplasia	
M	13.7	Cleft lip palate	
M	16.3	Agenesis of corpus callosum, cortical dysplasia, retrocerebellar arachnoid cyst	CGH array, LHX4, HESX1 negative

SOD: septo-optic dysplasia, ASD: atrial septal defect, VSD: ventricular septal defect

Genetic screening was performed in 25 syndromic patients (78%) and revealed three chromosomal abnormalities but no genetic abnormalities (Table 2); it was also performed in 10 nonsyndromic patients (29%) and was normal in all cases.

### Imaging evaluation

The MRI findings showed that neonatal patients more often had no visible (33% versus 2%; P = 0.0017) or a smaller (1.5 versus 2.8 mm; P = 0.0026) anterior pituitary lobe than patients with growth retardation (Table 1). Anterior pituitary height SDS was positively correlated with IGF1 SDS (R = 0.151; P = 0.0143).

### Hormonal status

All patients had GHD at diagnosis. Seventy seven percent of patients had complete GHD and the median GH peak during hypoglycemia or under stimulation test was 1.4 μg/L. At diagnosis, GHD was isolated (i.e., IGHD) in 35 patients (52%) and associated with other hormonal deficiency (i.e., CPHD) in 32 patients (48%).

**Fig 1:**
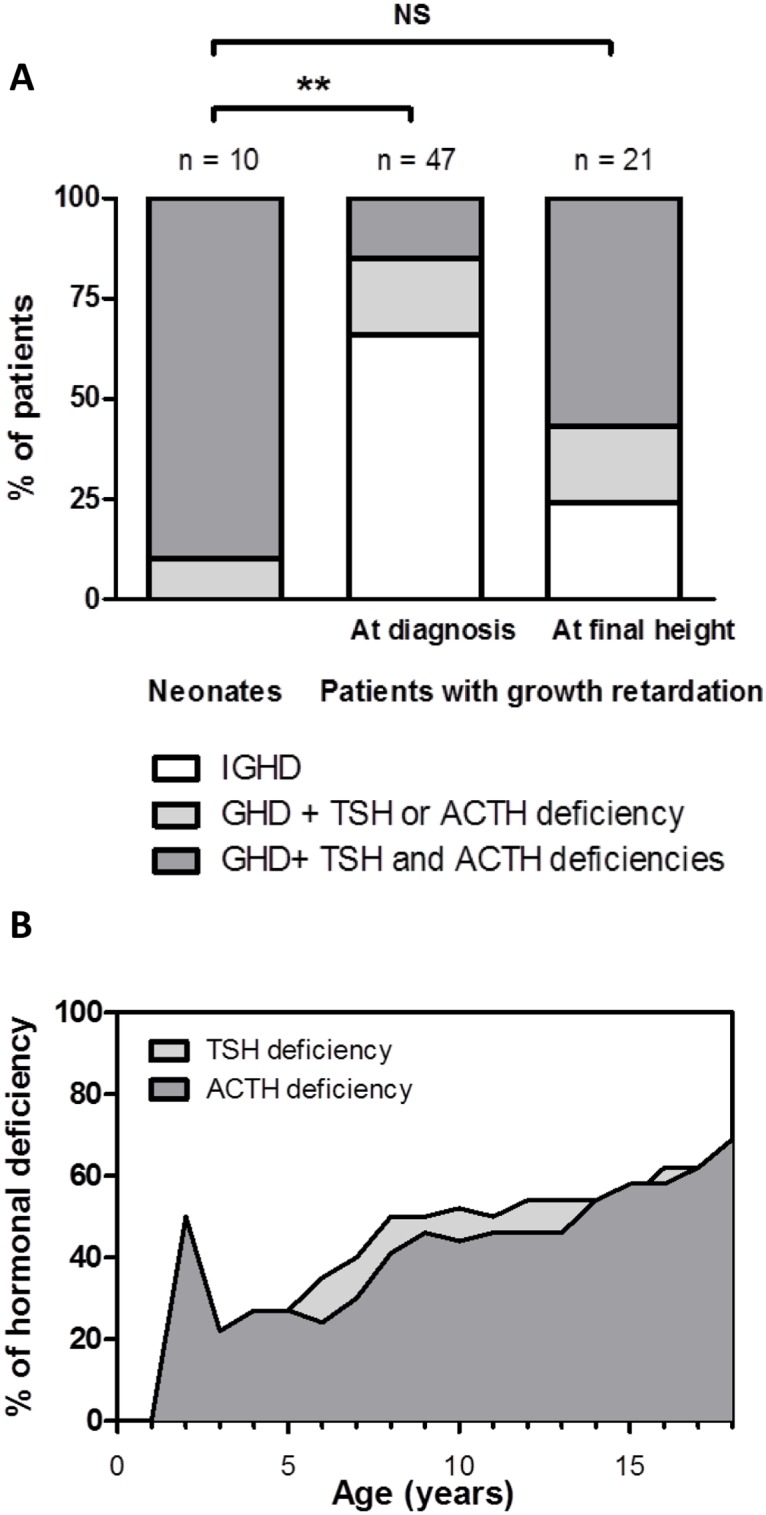
Evolution of the hormonal impairment according to the initial presentation. (A) Frequency of GH, ACTH and/or TSH deficiencies in neonates (n = 10) and patients with growth retardation, at baseline (n = 47) and at final height (n = 21). Significant statistical difference in the number of cases of combined pituitary hormone deficiency between groups of neonates and patients with growth retardation: ** P < 0.001 (chi square test). The gonadotropic axis was evaluated in neonates (during “mini-puberty”) and in patients of postpubertal age. LH/FSH deficiency was found in 9 of 10 neonates (90%) and 12 of 21 patients with growth retardation at final height (57%); this was not statistically different (P = 0.1522). (B) Evolution of ACTH, and TSH deficiencies throughout childhood in patients with growth retardation who reached their final height (n = 21). All patients had GHD at diagnosis.

The frequency of IGHD versus CPHD and the number of hormone deficits did not differ between the syndromic and nonsyndromic patients. In contrast, patients diagnosed in the neonatal period had greater hormonal impairment than growth retardation patients at diagnosis (Fig 1A). Thus, CPHD was found in all patients of the neonatal group and 34% of the patients with growth retardation (P = 0.0005). Interestingly, we were able to document a progressive worsening of the endocrine impairment in 21 patients of the growth retardation group followed until final height attainment, with a total follow-up duration ranging from 7.3 years to 16.6 years (median: 11.8 years) (Fig 1B). Reevaluation at completion of growth of these 21 patients showed that 17 patients (81%) presented with CPHD (Fig 1A); this frequency was no longer different from that of neonatal patients (P = 0.2043).

The gonadotropic axis was evaluated in neonates (during “mini-puberty”) and in patients of postpubertal age. LH/FSH deficiency was found in 9 of 10 neonates (90%) and 12 of 21 patients with growth retardation (57%); this was not statistically different (P = 0.1522).

Serum prolactin levels were available in 32 patients. They were low in 4 patients (12%) and increased in 14 patients (44%), suggesting a hypothalamic-pituitary disconnection (i.e., loss of normal dopaminergic inhibition of the PRL secretion). Only 3 patients (4%) presented with diabetes insipidus.

Multivariate studies showed that nonvisible anterior pituitary was an independent risk factor in terms of severity of hormonal impairment (P = 0.0342), but not gender or associated extra-pituitary malformations.

### Response to growth hormone treatment

**Fig 2:**
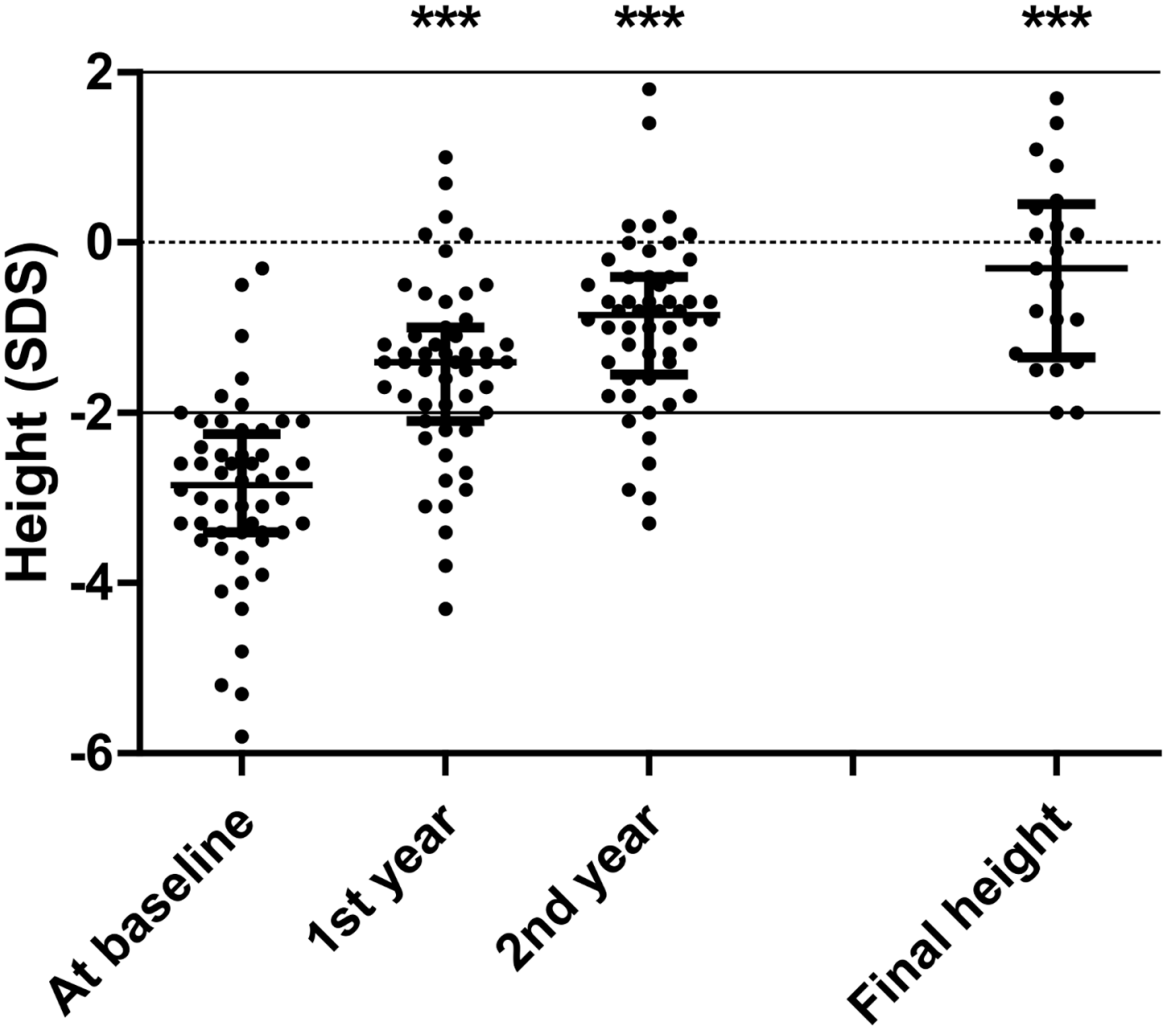
Height SDS during the first 2 years of GH treatment and at final height. Error bars are medians with interquartile ranges. Significant statistical differences between height SDS at baseline and others time points: *** P < 0.0001 (2-way repeated-measures ANOVA plus Bonferroni post-test).

Growth under GH treatment was similar in the groups with a median height gain SDS of 1.2 (range: -1.4 to 4.4 SDS) after one year and 1.7 (range: -0.4 to 3.0) after 2 years (Fig 2). For the 21 patients who reached their adult height, median height gain SDS was 2.3 (range: 0.7 to 5.4 SDS) with an adult height SDS of -0.3 (range: -2.0 to 1.7 SDS) and a difference from target height SDS of 0.2 (range: -2.7 to 1.4 SDS).

Multivariate studies showed that height SDS at baseline was negatively associated with height gain after 2 years of GH treatment. (R = -0.590; P = 0.0001), but not other determinants (age, gender, presence of malformation, nonvisible anterior pituitary lobe, and severity of hormonal impairment).

The adult height SDS was positively correlated with the target height (R = 0.516; P = 0.0184) and the height gain between diagnosis and adult height was negatively correlated with the height at baseline (R = -0.472; P = 0.0998).

## Discussion

In the present study, we described the clinical, hormonal and radiological characteristics at baseline and during long-term follow-up in a large cohort of patients with PSIS according to the initial presentation.

Only a few patients (15%) were referred for hypoglycemia during the neonatal period but interestingly they all presented with a particularly severe form of PSIS. Indeed, CPHD was found in all patients of the neonatal group and it was complete (i.e., associating GH, ACTH, TSH and LH/FSH deficiencies) in 9 of 10 patients. This severe phenotype was related to more severely abnormal pituitary development (i.e., anterior pituitary lobe not visible or smaller) than in patients diagnosed later on the basis of growth retardation. Abnormalities of the hypothalamic—pituitary region have been reported to be related to the severity of anterior pituitary dysfunction [2,3,13,14]. Similarly, we demonstrated that anterior pituitary lobe size was correlated with the IGF1 level and that a nonvisible lobe was an independent risk factor in terms of severity of hormonal status. In contrast, we found no correlation between anterior pituitary hormone deficiencies and the abnormality of the pituitary stalk.

As previously reported, most patients (70%) were initially brought to medical attention as children with growth retardation [24,25]. The median age at diagnosis of these patients was about 4 years, which is in accordance with published data [6,14,18,25–27]. Regular follow-up of growth might have allowed earlier diagnosis, as decreased growth velocity and growth retardation (i.e., height below -2 SDS) were present respectively 3 and 2 years before the referral. Moreover, features suggestive of neonatal hormonal deficiency (hypoglycemia, jaundice, and micropenis/cryptorchidism in males) were found in about one third of the patients in our study and others [14,18,24,27], and should therefore prompt earlier evaluation of pituitary function and morphology. The later diagnosis could be explained, at least in part, by less severe endocrine impairment. Thus, most patients diagnosed on growth retardation (66%) had isolated GHD at diagnosis. This might have caused moderate and spontaneously reversible hypoglycemia during the neonatal period (found in 14% of patients with growth retardation); however, we could not document the characteristics of hypoglycemia (i.e., severity, duration, and need for a treatment) in these patients.

The frequency of CPHD in PSIS patients varies among reports, ranging from half to all patients [18,27,28]. These discrepancies may be explained by differences in the age of patients at evaluation. Indeed, in contrast to patients diagnosed in the neonatal period, who present with complete hypopituitarism at diagnosis, most of the patients diagnosed after investigation for growth retardation presented a progressive impairment of the residual pituitary function throughout childhood. One patient in our cohort was diagnosed at the age of 16.3 years owing to moderately short stature (height SDS at -1.9) and delayed puberty. Similarly, Ioachimescu et al. reported the case of a 14-year-old girl diagnosed with PSIS on the basis of delayed puberty with normal height [17]. These findings underline the need for periodic follow-up assessment for potential additional hormonal deficiencies in patients with PSIS until adulthood.

Although malformation was the reason for referral in only 15% of patients, we found a high frequency of extra-pituitary malformations, especially in the central nervous system and the craniofacial structures, in accordance with previous reports [2,25,29,30]. This finding is in line with the hypothesis of abnormal pituitary development during the embryonic period and underlines the need for systematic screening for associated malformations. As expected, the presence of extra-pituitary malformations was associated with earlier diagnosis (4.1 years). Contrary to other studies [4,6], we did not find that the presence of these malformations was associated with more severe hormonal and radiological characteristics. Conversely, the diagnosis of PSIS should be considered in cases of malformation syndrome with short stature.

The male preponderance and the higher occurrence of breech delivery and cesarean section observed in our study are in keeping with other studies [2,4,14,16,18] and did not differ with the initial presentation.

Interestingly, growth under GH treatment was similar in the patient groups (neonates versus patients with growth retardation, and syndromic versus nonsyndromic patients) and did not vary according to the pituitary MRI findings. The long-term results for adult height were satisfactory with a total height gain SDS of 2.3 and a normalized adult height, which is comparable to the results of other studies [18,27,28]. As previously described, height SDS at baseline was a significant independent predictive factor of short- and long-term GH response, with short height at baseline showing a better response to treatment [30].

## Conclusion

The present study suggests that patients with PSIS diagnosed in the neonatal period have a particularly severe form of PSIS with greater hormonal impairment related to more severely abnormal pituitary development, and they require early diagnosis and treatment.

The high frequency of associated extra-pituitary malformations in patients with PSIS underlines the need for systematic screening for associated malformations. However, the presence of these malformations does not seem to be associated with more severe hormonal and radiological characteristics. Last, we documented a progressive worsening of endocrine impairment throughout childhood, justifying periodic follow-up to check for additional hormonal deficiencies in patients with PSIS.
